# Effectiveness of bitter melon extract in the treatment of ischemic wounds in rats

**DOI:** 10.3906/biy-1804-36

**Published:** 2018-12-10

**Authors:** Öznur GÜRLEK KISACIK, Ülkü GÜNEŞ, Mustafa Volkan YAPRAKÇI, Korhan ALTUNBAŞ

**Affiliations:** 1 Department of Basic Nursing, Faculty of Health Science, Afyonkarahisar Health Science University , Afyonkarahisar , Turkey; 2 Department of Basic Nursing, Faculty of Nursing, Ege University , Bornova, İzmir , Turkey; 3 Department of Histology and Embryology, Faculty of Veterinary Medicine, Afyon Kocatepe University , Afyonkarahisar , Turkey; 4 Department of Surgery, Faculty of Veterinary Medicine, Afyon Kocatepe University , Afyonkarahisar , Turkey

**Keywords:** Ischemic wound, bitter melon, Momordica, wound dressing

## Abstract

There is no consensus on the properties of an ideal dressing for treating wounds. The aim of this study was to investigate the efficacy of dressings using topically administered bitter melon extract with olive oil, pure olive oil, nitrofurazone, and saline in the healing of ischemic wounds. A sample group of 48 rats was used in the trial. Their wounds were treated with bitter melon extract, pure olive oil, nitrofurazone, and saline. Data were collected between October 2014 and April 2015. The highest percentage (94.7%) of wound healing was observed in the bitter melon extract group and the lowest percentage (86.3%) in the nitrofurazone group. At the end of the 21st day, macroscopic reepithelialization was observed in 9 wounds in the bitter melon extract group (75%), in 6 wounds in the pure olive oil group (50%), and in only 3 wounds in the nitrofurazone and saline groups (25%). It can be concluded that dressing with a bitter melon extract is more efficient in the treatment of wounds than using nitrofurazone or saline, and that dressing with olive oil accelerates wound healing, although not as much as dressing with bitter melon extract.

## 1. Introduction

The treatment of wounds is one of the oldest topics of discussion and has been studied for many years. New products related to the care of wounds are constantly being developed, in parallel to continual advances in medicine. Although there are many dressing products currently on the market, their high costs are a heavy economic burden on both individual patients and nation states. Furthermore, there is no consensus on the properties an ideal dressing should have for treating wounds (Dorai, 2012; Maver et al., 2015; Pereira and Bártolo, 2016) .

In developing countries, 80% of people use conventional medicine to treat their health problems (Dandawate et al., 2016) . As a result of this, many recent studies have focused on what kinds of effective and safe therapeutic agents can be obtained from natural sources to treat different diseases (Palamthodi and Lele, 2014; Agyare et al., 2016; Jarić et al. 2018) . Various herbs have been used to treat wounds due to their availability and the low risk of side effects (Kumar et al., 2013; Budovsky et al., 2015) . Bitter melon extract is one of the herbs most frequently used in Turkey for its medicinal properties (Pişkin et al., 2014; Dandawate et al., 2016) .

Bitter melon extract, which is formed by incubating the herb in olive oil, has been used externally for wound care and orally for the treatment of stomach complaints caused by peptic ulcers (Wang et al., 2017) . Recent studies conducted on the pharmacological properties of bitter melon demonstrated that this plant has antidiabetic, antilipidemic, antioxidant, antibacterial, antiinflammatory, antiviral, and anticancer activities (Prashanthi et al., 2012; Nagarani et al., 2014; Panda et al., 2015; Raina et al., 2016; Mahmoud et al., 2017; Rashid et al., 2017; Saad et al., 2017) . However, there is as of yet a limited number of studies investigating its effects on healing wounds.

Bitter melon (Momordica charantia) is a thin, ascending, ivy-like vine that flowers once a year and is a member of the family Cucurbitaceae. It is cultivated for medicinal purpose and includes many biologically active compounds. Among these, momordicin, momorcharin, momordin, charantin, polipeptide-p, and cucurbitacin B are the main substances that it contains. Bitter melon also contains high amounts of vitamin C. The fruit and leaves of the plant are rich in minerals and vitamins, and it is an important source of iron, calcium, magnesium, phosphorus, and vitamin B. Furthermore, it is known to contain β-carotene, potassium, vitamin A, and zinc (Zhang et al., 2016; Jabeen et al. 2017; Jia et al., 2017) . There are many studies in the literature demonstrating the conventional use of bitter melon as a preventive and therapeutic herb for a large range of diseases, and it contains a number of different biological chemical compounds (Ahmad et al., 2012; Hamissou et al., 2013; Talukder et al., 2013; Raina et al., 2016) .

The mature fruits of the plant have been used in wound care. Studies investigating the efficacy of the topical administration of bitter melon extract have demonstrated that the plant has a significant and effective role in wound healing due to its wide-spectrum antibacterial, antioxidant, antiinflammatory, analgesic, and antiulcer properties, and because it enhances the activities of transforming growth factor beta (TGF-β) (Prashanthi et al., 2012; Jung et al., 2013; Hussan et al., 2014; Alippilakkotte et al., 2017; Singh et al. 2017) .

One of the most commonly known properties of bitter melon is its capacity for healing wounds. It has been reported that within the process of wound healing, the plant accelerates the production of growth factors, induces the proliferation of fibroblasts, and increases the oxygenation of the wound, an important factor in healing, by accelerating capillary circulation; it also accelerates the process of healing due to the antioxidant and antimicrobial effects of the phytochemical substances such as flavonoids and glycosides that it contains, and it positively effects the rate of wound healing, the ability of the wound to contract, the time for the wound to close, the epithelization process, and the tension of the wound (Teoh et al., 2009; Süntar et al., 2010; Prashanthi et al., 2012; Satar et al., 2013; Supraja and Usha, 2013; Pişkin et al., 2014; İlhan et al., 2015; Singh et al., 2017) .

The aim of this study was to investigate the wound healing effects of bitter melon extract, and for this purpose we compared its effects with those of pure olive oil, nitrofurazone, and saline in the treatment of ischemic wounds.

## 2. Materials and methods

### 2.1. Sample

This study was performed in the Experimental Laboratory Animals Application and Research Center, Afyon Kocatepe University, between 15.10.2014 and 01.04.2015. The sample of the study comprised 48 male Wistar rats 1.5–2 months old and weighing 200–250 g, selected through a simple randomized sampling method. The power analysis was calculated as 0.80 with a 0.50 effect size when 12 animals were studied in each group.

The health of the animals used in the study was checked by a veterinarian in the Experimental Laboratory Animals Application and Research Center. Due to the effect of rats potentially dying before the completion of 21 days of treatment and follow-up, and because the effect of the reproductive cycle on the healing process could not be precisely deduced, female rats were excluded from the study.

### 2.2. Randomization

**Figure 1:**
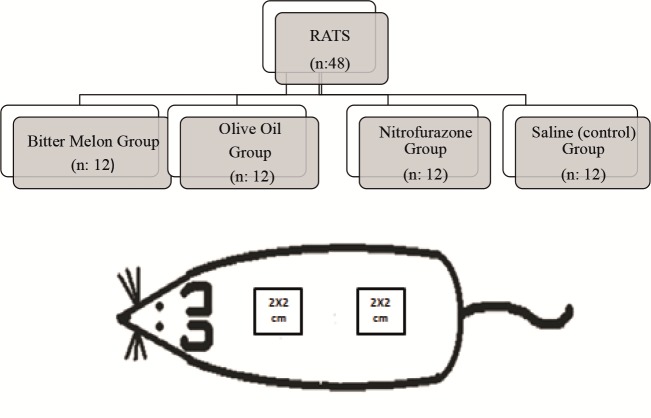
Flowchart and schematics. The top image is a randomization flowchart. The bottom image is a schematic figure of the wounds to the rats.

The rats were classified into the treatment and control groups via a simple randomization method using a random number generator. The treatment groups were the bitter melon group (Group A), the pure olive oil group (Group B), the nitrofurazone group (Group C), and the saline (control) group (Group D). A total of 48 rats were randomly assigned to the treatment and control groups according to the order determined by 12 randomly obtained number combinations (12, 13, 24, 7, 10, 4, 13, 20, 22, 19, 8, 15) (Figure 1). The rats were kept at a room temperature of 21 °C with a light/dark cycle of 12 h. Each rat was kept in single cage, fed ad libitum with pellet feed and water, and allowed to move freely within the cage.

### 2.3. Treatment materials

The number of large-scale clinical trials on how to prepare and use bitter melon is insufficient. However, for traditional wound healing, information on how to prepare the extract can be found in studies that examine the effectiveness of bitter melon extract with olive oil (Satar et al., 2013). In our study, the bitter melon extract with olive oil was prepared according to the recommended preparation method for traditional wound healing as stated in the literature (Satar et al., 2013). In addition, advice was sought from the Department of Pharmacognosy in the Faculty of Pharmacy at Ege University. The extract obtained was kept in a refrigerator until the moment of use.

To produce the bitter melon extract, the mature fruits of bitter melons harvested from Bursa in August were cut open with a knife, the red seeds of the fruits were removed, and the fruit was then cut into small pieces. The fruit (100 g) was then placed into glass jars containing 500 mL of pure olive oil. The jars were incubated under sunlight for 1.5 months and then homogenized via a sterile spatula to form a pulp. Nitrofurazone (0.2%, 56 g) ointment was obtained from a local pharmacy and used in this study. Extra virgin olive oil from a local market was used as a pure olive oil with an acidity degree up to 0.8%.

### 2.4. Ethical considerations

Forty-eight rats were used in the animal experiments following the Guidelines for the Care and Use of Laboratory Animals and as approved by the Animal Studies Local Ethics Committee of Afyon Kocatepe University (49533702/97).

### 2.5. Primary outcomes

The primary outcomes of the study were wound healing percentages, macroscopic reepithelialization, and histological examination criteria from day 1 to day 21.

### 2.6. Data collection

Bitter melon extract was obtained from plants from a single region. The ischemic wound model was created on the back of each rat by a veterinarian using a bipedicle flap. Two full layer wounds of 2 × 2 cm were created within the ischemic area on the 3rd postoperative day. These wounds were dressed each day for 21 days for each group of rats.

### 2.7. Ischemic wound creation

In this study, bipedicle flaps were created on the back of the rats to induce ischemia. Prior to the surgical procedure, ketamine (75–100 mg/kg) and xylazine (1–5 mg/kg) were administered through the intramuscular route to anesthetize each rat. The hair in an area of 4 × 10 cm between the scapulae and the iliac processes was softened with surgical soap and shaved. The rats under anesthesia were laid in the ventral recumbent position (backs up, abdomens down) and stabilized. The area of 4 × 10 cm was marked with a sterile marker pen. Each mark was checked with a sterile millimetric ruler.

The surgical area was cleaned with antiseptic solution and the two parallel edges from the scapulae to the iliac processes were cut with a lancet and tissue scissors. Flap subcutaneous tissue was then dissected including the panniculus carnosus (the subcutaneous structure providing vascularization). The flap was replaced and sutured using 3/0 silk with 1-cm intervals.

In order to provide standardization of the wound on the back of each rat, a model was created on millimetric acetate paper. The model was drawn on this paper so as to produce square wounds of 2 × 2 cm at a 1-cm distance from the flap edges in the horizontal position and at a 2-cm distance in the vertical position, one in the cephalic and one in the caudal positions. Each acetate paper with the wound model was sterilized using ethylene oxide. The rats with bipedicle flaps were left unattended for the first two postoperative days, and the wounds began to form on the 3rd day. Prior to the procedure the skin was cleansed with antiseptic solution. The procedure was performed under sterile conditions and the sterile wound model was placed on the bipedicle flap area; the borders of the area were marked with a sterile marker pen. A total of two fulllayer wound defects of 2 × 2 cm were formed on the back of each rat with the help of a lancet and tissue scissors. The wounds were 1 cm distant from each other in the horizontal position and 2 cm apart in the vertical position.

### 2.8. Wound treatment protocol

The wounds of the rats were treated with bitter melon extract with olive oil, pure olive oil, nitrofurazone, or saline for 21 days. The wound areas were cleansed with sterile saline prior to being dressed. Due to the different forms and densities of the substances used in the study and control groups, in order to standardize the dressing methods the lowest amount that would fill the wound area was used and this amount was applied with the help of a sterile spatula to form a thin film layer. One of 3 g of bitter melon extract, 1 mL of pure olive oil with a maximum acidity of 0.8%, or 1 g of nitrofurazone pomade was administered onto the wounds of the rats according to the groups they were assigned to. Sterile saline was administered only to the rats in the control group. The wounds were closed with a sterile sponge and the adhesive bandage was stabilized with the dressing. The wounds of each rat were dressed once a day in all the groups.

### 2.9. Wound healing rates

The unhealed wound area and the percentage of total wound healing were recorded on each day of measurement and used for statistical analysis. On the 7th, 14th, and 21st days the wounds were drawn on millimetric acetate papers. These were then scanned and transferred to electronic form. The wound area was calculated using Auto CAD R14 (Autodesk Inc., San Rafael, CA, USA) software. The following formula was used to calculate the rate of wound closure (Walker, 1969) :

Walker's formula:

$\mathrm{Wound area}\%=\frac{\mathrm{Wound area on day \times}}{\mathrm{Wound area measured on day 1}}x100$

Wound healing percentage = 100 – percentage of the wound area

### 2.10. Determination of wound healing

The bandages were changed daily throughout the 21 days and medication was applied each time this occurred. Each wound was evaluated for the presence of exudate or other abnormalities and for wound appearance when the bandages were changed. The macroscopic reepithelization criteria of Geronemus et al. (1979) were used for determining the day of full healing of the wound (pink appearance on the wound without scab and complete closure of the wound). Macroscopic reepithelization was evaluated as “present” or “absent” on days 7, 14, and 21.

### 2.11. Histological examination

Following the evaluation of the wounds on day 21, the rats received high-dose anesthesia and were then exterminated. Excisional biopsy was performed on the tissue samples including the edges and the surface of each wound for histological examination. The tissues were fixed in 10% neutral formalin for 2 days. They were then washed and dehydrated by incubating them in increasing concentrations of alcohol.

They were rendered transparent in xylol and embedded in parafin. Sections of 5 µm were obtained using a Leica RM 2125 RT. The sections were then stained using Crossman’s modified triple staining method. The slides were examined under an Olympus BX50 microscope with regard to epithelialization, collagen, fibroblasts, inflammatory cells, and new vessel formation. No finding (-) was scored as 0, partial/poor (+) was scored as 1, completed but immature or mild (++) was scored as 2, completed and mature/ moderate (+++) was scored as 3, and significant (++++) was scored as 4. Images were recorded using an Olympus DP 25 camera.

### 2.12. Statistical analysis

Analysis of the study data was carried out using SPSS 20.0 (IBM Corp., Armonk, NY, USA). The descriptive statistics of continuous variables were expressed as mean, standard deviation (SD), median, and minimum and maximum values, and the descriptive statistics of the categorical variables were expressed as frequency and percentages. The suitability of the continuous variables to normal distribution was investigated with the Shapiro–Wilk test. The Kruskal–Wallis test was used for the comparison of the four independent groups including nonnormally distributed variables, and the Bonferroni corrected Mann– Whitney U test was used for the subgroup comparisons. The differences between the dependent groups (differences between measurements at different times) were compared using Wilcoxon’s test. Pearson’s chi-square test was used for the intergroup comparison of the categorical variables. P < 0.05 was accepted as statistically significant.

## 3. Results

The mean weights of the rats were 245 ± 9 g at the beginning of the study, 238 ± 21 g on day 7, 251 ± 23 g on day 14, and 264 ± 25 g on day 21.

### 3.1. Wound healing percentages

**Figure 2:**
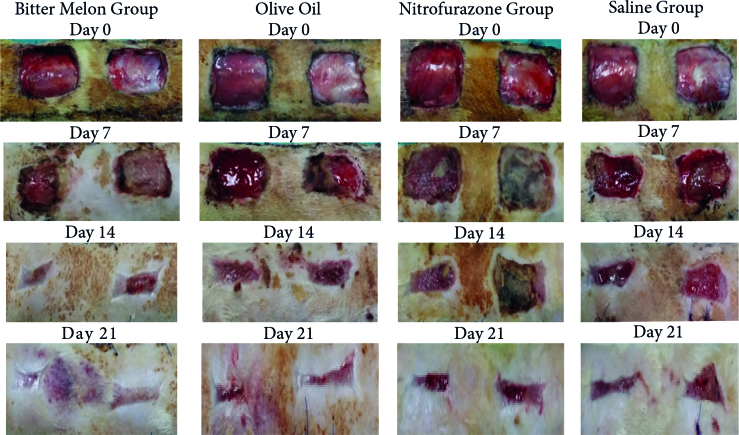
Progression of wound healing in the bitter melon, olive oil, nitrofurazone, and control groups from day 0 to day 21.

**Figure 3:**
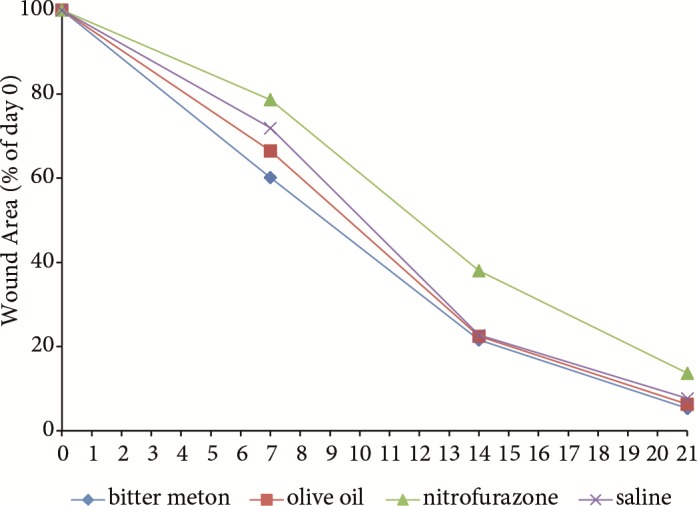
Quantitative analysis of wound areas per group, expressed as percentage of the initial wound size at day 0.

Table 1 shows the healing percentages of all groups on days 7, 14, and 21. The group with the highest percentage of healing was bitter melon and the lowest was nitrofurazone. A significant difference was observed between the groups with regard to the healing percentages on days 7 and 14 (respectively P = 0.022 and P = 0.003). Although the wound healing percentage on day 7 and 14 in the bitter melon group was higher than that of the nitrofurazone and saline (control) groups (P = 0.004), no significant difference was observed compared to the olive oil group (P > 0.05). However, the difference between the groups was not significant on day 21 (P = 0.052) (Table 1; Figures 2 and 3).

**Table 1:** Comparison of the mean unhealed wound areas and healing percentages of the wounds in the treatment and control groups.

Group	Day 0	Day 7		Day 14		Day 21	
	Wound area (cm2)	Unhealed wound area (cm2)	Total wound healing (%)	Unhealed wound area (cm2)	Total wound healing (%)	Unhealed wound area (cm2)	Total wound healing (%)
		Mean ± SD (cm2) Median (min-max)	Mean ± SD (cm2) Median (min-max)	Mean ± SD (cm2) Median (min-max)	Mean ± SD (cm2) Median (min-max)	Mean ± SD (cm2) Median (min-max)	Mean ± SD (cm2) Median (min-max)
Bitter Melon	4.00 ± 0	2.40 ± 0.63a 2.33 (1.60–3.34)	39.9 ± 15.6a 41.62 (16.37–60.00)	0.86 ± 0.46a 0.67 (0.33–1.84)	78.4 ± 11.4a 83.12 (64.0–91.62)	0.22 ± 0.23 0.14 (0–0.64)	94.7 ± 5.8 96.56 (84.0–100.0)
Olive Oil	4.00 ± 0	2.67 ± 0.63a 2.52 (1.81–3.86)	33.5 ± 15.8a 37.44 (3.37–54.62)	0.88 ± 0.53a 0.67 (0.33–1.84)	77.5 ± 13.2a 78.94 (52.5–95.5)	0.25 ± 0.24 0.21 (0–0.68)	93.7 ± 6.0 94.81 (82.87–100.0)
Nitrofurazone	4.00 ± 0	3.14 ± 0.31b 3.16 (2.60–3.61)	21.4 ± 7.7b 20.87 (9.62–35.0)	1.52 ± 0.38b 1.43 (0.85–2.18)	62.0 ± 9.5b 64.19 (45.5–78.62)	0.54 ± 0.36 0.62 (0.04–1.06)	86.3 ± 8.9 84.44 (73.37–99.0)
Saline (control)	4.00 ± 0	2.68 ± 0.40b 2.68 (1.98–3.34)	28.0 ± 14.1b 30.88 (3.75–50.5)	0.91 ± 0.30b 0.97 (0.43–1.49)	77.2 ± 7.6b 75.75 (62.75–89.25)	0.31 ± 0.17 0.31 (0–0.65)	92.3 ± 4.4 92.25 (83.75–100.0)
χ2*, P-value		χ2 = 9.936, P = 0.022*		χ2 = 14.084, P = 0.003*		χ2 = 7.731, P = 0.052	

*Kruskal–Wallis test, P < 0.05. a,b Different superscripts within the same column indicate significant difference among groups.

### 3.2. Macroscopic reepithelialization

No macroscopic reepithelialization (complete healing and closure of the wound) was observed in the wounds of the treatment and saline (control) groups on days 7 and 14, whereas it was observed in 9 wounds (75%) in the bitter melon group, in 6 wounds (50%) in the olive oil group, and in 3 wounds (25%) in the nitrofurazone and saline (control) groups on day 21, which was statistically significant (χ 2 = 3.641, P = 0.046).

### 3.3. Histological examination

**Figure 4:**
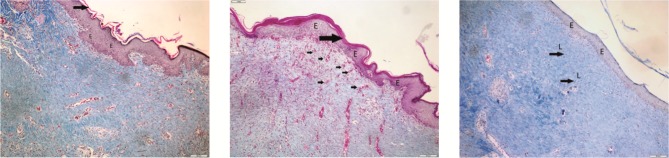
It was observed that fibrosis occurred by completing the epithelialization, new vessel formation was significant, and leukocyte infiltration was low in the section taken on day 21 in the bitter melon group. Epidermis layer (E), keratin layer (large arrow), blood vessel wall (small arrow), leukocyte infiltration (L); Crossman’s modified triple staining, 1 bar = 200 μm.

**Figure 5:**
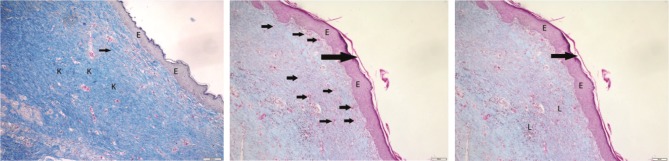
It was observed that epithelialization was completed, collagen formation was significant, new vessel formation was moderate, and leukocyte infiltration was low in the section taken on day 21 in the olive oil group. Epidermis layer (E), keratin layer (large arrow), blood vessel wall (small arrow), collagen formation (K), leukocyte infiltration (L); Crossman’s modified triple staining, 1 bar = 200 μm.

**Figure 6:**
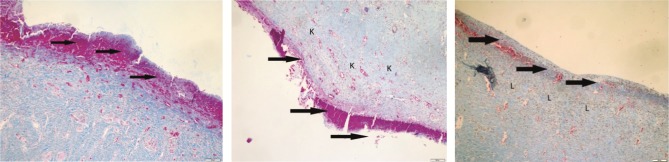
It was observed that epithelialization was incomplete, collagen formation was mild, and leukocyte infiltration was moderate in the section taken on day 21 in the nitrofurazone group. Incomplete epithelialization (thick arrow), mild degree of collagen formation (K), leukocyte infiltration (L); Crossman’s modified triple staining, 1 bar = 200 μm.

**Figure 7:**
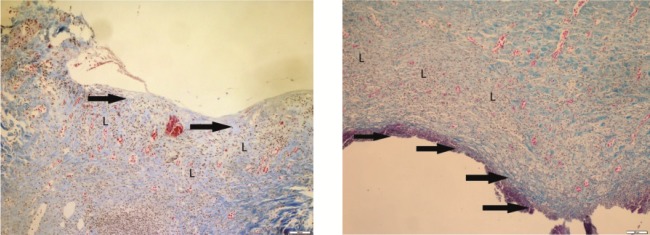
It was observed that epithelialization had not occurred and leukocyte infiltration was significant in the section taken on day 21 in the control group. Incomplete epithelialization (thick arrow), significant level of leukocyte infiltration (L); Crossman’s modified triple staining, 1 bar = 200 μm.

When the wounds were evaluated according to the histological examination criteria, the highest epithelialization rate was observed in the bitter melon extract group (2.29 ± 1.64), which was followed by the olive oil group (1.83 ± 1.57), on day 21. Histological examinations revealed the highest collagen formation rate in the olive oil group (3.08 ± 0.60), which was followed by the bitter melon group (2.92 ± 0.67). The collagen formation rate was lowest in the nitrofurazone group (2.29 ± 0.50). Fibroblast formation was significantly higher in the bitter melon (3.38 ± 0.59) and olive oil (2.75 ± 0.50) groups, whereas it was low in the saline (control) and nitrofurazone groups, and this was found to be a statistically significant difference (P < 0.001). Inflammatory cell formation was highest in the saline (control) group (2.75 ± 0.84) and lowest in the bitter melon group (2.25 ± 0.97); however, no difference was observed between the groups with regard to mean inflammatory cell scores (P = 0.354). New vessel formation was highest in the bitter melon group (3.54 ± 0.62); however, the difference between the groups was not statistically significant (P = 0.124) (Table 2; Figures 4-7).

**Table 2:** Comparison of the mean histological scores of the wounds in the treatment and control groups.

	Bitter melon	Olive oil	Nitrofurazone	Saline (control)		
Histological scores	Mean ± SD Median (min–max)	Mean ± SD Median (min–max)	Mean ± SD Median (min–max)	Mean ± SD Median (min–max)	χ2* value	P-value
Epithelialization scores	2.29 ± 1.64 2.00 (0–4.0)	1.83 ± 1.57 2.00 (0–4.0)	0.83 ± 1.03 0 (0–2.0)	0.21 ± 0.58 0 (0–2.0)	17.082	0.001*
Collagen scores	2.92 ± 0.67 2.75 (2.0–4.0)	3.08 ± 0.60 3.00 (2.0–4.0)	2.29 ± 0.50 2.0 (1.5–3.0)	2.67 ± 0.44 3.0 (2.0–3.0)	10.546	0.014*
Fibroblast scores	3.38 ± 0.59 3.50 (2.5–4.0)	2.75 ± 0.50 2.75 (2.0–3.5)	2.08 ± 0.42 2.0 (1.5–3.0)	2,25 ± 0,45 2.0 (1.5–3.0)	24.742	0.000**
Inflammatory cell scores	2.25 ± 0.97 2.00 (1.0–4.0)	2.33 ± 0.81 2,50 (1,0–4,0)	2.58 ± 0.56 2.5 (1.5–3.5)	2.75 ± 0.84 2.5 (1.5–4.0)	3.253	0.354
New vessel scores	3.54 ± 0.62 3.75 (2.0–4.0)	2.92 ± 1.06 3.25 (1.0–4.0)	3.29 ± 0.69 3.5 (2.0–4.0)	2.75 ± 0.94 2.75 (1.0–4.0)	5.754	0.124

*Kruskal–Wallis test, P < 0.05, **P < 0.001.

## 4. Discussion

Wound healing is a complex process that comprises three phases of inflammation, proliferation, and maturation, and it involves the well-organized and highly complex interaction of different tissues and cells (Agyare et al., 2016;

and leukocyte infiltration was low in the section taken on day 21 in the olive oil group. Epidermis layer (E), keratin layer (large arrow), blood vessel wall (small arrow), collagen formation (K), leukocyte infiltration (L); Crossman’s modified triple staining, 1 bar = 200 µm. Jarić et al., 2018 ). Preliminary phytochemical screening 2014; İlhan et al., 2015; Zhang et al., 2016; Jia et al. 2017). of the extracts of bitter melon showed that there were Previous studies revealed the wound healing activity tannins, alkaloids, flavonoids, glycosides, and saponins of bitter melon, and the promotion of wound healing in the extract. Tannins are known to have antimicrobial, through antiinflammatory and antibacterial effects and astringent, and protein coagulatory properties that aid the stimulation of angiogenesis and fibroblast activity in wound healing (Agyare et al., 2014; Nagarani et al., has been demonstrated in animal studies (Ilango et al.,

2010; Prashanthi et al., 2012; Satar et al., 2013; Hussan et al., 2014; Pişkin et al., 2014; İlhan et al. 2015; Singh et al. 2017). Furthermore, in a different study, it was reported that bitter melon fruit extract-based PLA/Ag nanobfiers used as wound healers can enhance the proliferation and function of epidermal cells and fibroblasts (Alippilakkotte et al., 2017) .

Measurement of the wound area and change in the surface area of the wound provides objective evidence of the wound healing process. In this study, the wound closure rate in the bitter melon group was 1.5 times greater than that of the olive oil group and 3 times greater than that of the nitrofurazone and the saline (control) groups. The increased epithelialization, fibroblast density, and new vessel formation in the bitter melon group revealed in histological examinations also confirmed this difference. The literature on the positive efficacy of bitter melon on wound healing is promising. In a study on rats, the wound closure rates in the group treated with bitter melon were twice as fast as those of the control group. Additionally, the hydroxyproline level in granulation tissue, which is an important indicator of the wound healing process and is used as a marker of the amount of collagen in tissue, was reported to be higher in the bitter melon group (Prashanthi et al., 2012) . Likewise, in another study, the olive oil macerate of bitter melon showed significant wound healing activity both in incision and excision wounds and significant enhancement in hydroxyproline content in buccal mucosa wounds in rats (İlhan et al., 2015) .

Wound closure is influenced by the formation and maturation of collagen. In our study, the highest epithelialization was observed in the bitter melon group, and collagen formation was higher in the olive oil group.

In a similar study conducted on rabbits, the wounds were treated with bitter melon extract, olive oil, and a no-treatment protocol. The wounds were observed to be completely closed in the bitter melon group on day 28, and the time for complete closure was significantly shorter compared to the pure olive oil group (Satar et al., 2013).

In another study, wounds formed in rabbits were treated with dexpanthenol, bitter melon, and nitrofurazone.

Mild collagen fiber formation and a high rate of vessel formation were observed in the group treated with bitter melon ( Pişkin et al., 2014 ). Singh et al. (2017) reported that topical extracts of bitter melon can increase the formation of granulation tissue. Hussan et al. (2014) reported that bitter melon ointment can accelerate ulcer healing and increase TGF-β 52 expression. In another study conducted by Agyare et al. (2014) it was found that bitter melon extract increased collagenation in the wound tissue and the rate of wound closure. Our results also support the literature.

Antiinflammatory activity is essential for the wound healing process, since long periods in the inflammatory phase results in retardation of healing. Administration of antiinflammatory and antioxidant agents may be beneficial in healing skin wounds (İlhan et al., 2015) . It has been reported that bitter melon has higher potential antioxidant and antiinflammatory activities (Nagarani et al., 2014; Aljohi et al., 2016; Wang et al., 2017) . Likewise, in the study conducted by İlhan et al. (2015), bitter melon extract showed significant wound healing and antiinflammatory effects. In our study, inflammatory cell formation was lowest in the bitter melon group. The efficacy of bitter melon on wound healing may be related to its antiinflammatory effects, which reduce the bacterial load of the wound and increase epithelial proliferation.

Furthermore, bitter melon contains high amounts of vitamin A, which is necessary for epithelial keratinization and formation of collagen cross-fibers; vitamin C, which is a strong antioxidant; and zinc, which is known to play a key role in wound healing. The high collagen formation scores observed in the wounds in the bitter melon group in this study are consistent with findings in the literature (Prashanthi et al., 2012; Agyare et al., 2014) .

Oxidative stress, known to be caused by excessive oxidants, is very important in wound healing because it causes further damage to tissues and therefore reduces or delays the healing process. It has been reported in the literature that the analgesic and antiinflammatory activities of bitter melon accelerate capillary circulation, thereby increasing oxygenation, which is an important factor in wound healing, and that the antiinflammatory and antioxidant activities reduce the harm caused by free radicals formed as a result of the inflammation and prevent the progression of necrosis (Kumar and Bhowmik, 2010; Agyare et al., 2014; Aljohi et al., 2016; Rosyid et al., 2018) .

The optimum environment for epithelialization is a moist environment. Moreover, it has been reported that the hypoxic environment under moist wound dressings increased capillary proliferation; angiogenesis is faster in moist conditions (Boateng and Catanzano, 2015) . In an in vitro study conducted by Aljohi et al. (2018) , it was reported that the methanolic extract of bitter melon had angiogenic effects. Singh et al. (2017) reported that extracts of bitter melon topically can increase angiogenesis. Similar to the literature, in our study it was found that new vessel formation was highest in the bitter melon group. These observations support the beneficial effects of topical bitter melon extracts on wound healing.

The most obvious finding to emerge from this study is that the healing rate observed in the bitter melon group was significantly faster than that seen in the nitrofurazone and the saline (control) groups, and that the healing rate in the olive oil group was close to the one observed in the bitter melon group. The promising outcomes observed in wound healing using bitter melon extract could form a basis for controlled clinical studies. In this respect, since preparation of the bitter melon and pure olive oil is easy and the economic aspects are favorable, it could be used as an alternative treatment option in the treatment of wounds. Controlled clinical studies should be conducted on individuals with chronic wounds that are difficult and time-consuming to treat.

However, this study has some limitations. This was an in vitro study. One limitation of this study was that it was not a clinical study performed on humans due to time, ethical concerns, and budget. Another limitation was that it was not a blinded study due to its design. In addition, we know that there are many factors that can effect wound healing and that may interfere with one or more phases in this process. In this study, we were not able to examine and control all these factors, and they may have affected our results. Furthermore, the 21-day follow-up was also a limitation. We could not investigate the biochemical pathways through which the bitter melon has its healing effect nor analyze the active substances in the plant extract due to our limited budget.

## Acknowledgment

This study was funded by the Scientific and Technological Research Council of Turkey (TÜBİTAK) as part of the 1002-Fast Foundation Project with number 1150929.
